# 133. ARGONAUT-III: Susceptibility of Carbapenem-resistant *Klebsiellae* to Cefepime-Taniborbactam

**DOI:** 10.1093/ofid/ofab466.133

**Published:** 2021-12-04

**Authors:** Andrew R Mack, Christopher Bethel, Steven Marshall, Robin Patel, Robin Patel, David van Duin, Vance G Fowler, Daniel D Rhoads, Michael Jacobs, Focco van den Akker, David A Six, Greg Moeck, Krisztina M Papp-Wallace, Robert A Bonomo

**Affiliations:** 1 Case Western Reserve University & Louis Stokes Cleveland VA Medical Center, Cleveland, Ohio; 2 Louis Sokes Cleveland VA Medical Center, Cleveland, OH; 3 Louis Stokes Cleveland Medical Center, Cleveland, OH; 4 Mayo Clinic, Rochester, MN; 5 University of North Carolina, Chapel Hill, North Carolina; 6 Duke University, Durham, North Carolina; 7 Cleveland Clinic, Cleveland, Ohio; 8 University Hospital Cleveland Medical Center, Cleveland, OH; 9 Case Western Reserve University, Cleveland, Ohio; 10 Venatorx Pharmaceuticals, Inc., Malvern, Pennsylvania; 11 Venatorx Pharmaceuticals, Malvern, Pennsylvania; 12 Louis Stokes Cleveland VAMC and Case Western Reserve University, Cleveland, OH; 13 Louis Stokes Cleveland VA Medical Center, Cleveland, OH

## Abstract

**Background:**

*Klebsiellae* are Gram-negative pathogens responsible for serious nosocomial and community-acquired infections. Carbapenem resistance, both intrinsic and acquired, complicates therapy. Taniborbactam (formerly VNRX-5133; Fig 1) is a bicyclic boronate β-lactamase inhibitor (BLI) that inhibits all four Ambler classes of β-lactamase enzymes, both serine- and metallo-, with the notable exception of class B IMP β-lactamases. Taniborbactam is currently undergoing phase 3 clinical trials in combination with cefepime (FEP; Fig 1) as part of the β-lactam-BLI (BL-BLI) combination FEP-taniborbactam (FTB).

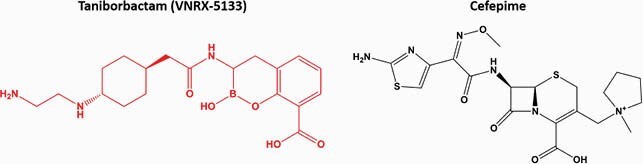

Figure 1. Structures of taniborbactam and cefepime. The β-lactamase inhibitor is in red and the β-lactam antibiotic is in black.

**Methods:**

We determined the activity of FTB against 200 carbapenem-resistant *Klebsiellae* (CRK) strains collected as part of the Antibiotic Resistance Leadership Group (ARLG) Consortium on Resistance against Carbapenems in *Klebsiella* (CRACKLE) study. Among these strains, 193 expressed class A KPCs, one expressed a class B NDM, and six expressed class D OXA-48 or variants. Broth microdilution minimum inhibitory concentrations (MIC)s were determined using the ThermoFisher Sensititre system with custom assay panels. American Type Culture Collection strains were used for quality control. The susceptible-dose-dependent breakpoint for FEP was provisionally used for FTB, where taniborbactam was fixed at 4 µg/mL.

**Results:**

Among the 200 *Klebsiella* strains tested, susceptibility for β-lactams alone ranged from 1% for ceftazidime (CAZ), 2.5% for meropenem, and 13.5% for FEP (Table 1). The addition of BLIs increased % susceptibility compared to BL alone to: 98% for CAZ-avibactam (CZA); 95.5% for MEM-vaborbactam (MVB); and 99.0% for FTB. MIC_50_ and MIC_90_ were in the susceptible and provisionally susceptible range for CZA and MVB, and in the provisionally susceptible range for FTB. Analyzing the CZA and MVB non-susceptible strains, 7 of 9 MVB non-susceptible strains and 2 of 4 CZA-resistant strains were provisionally susceptible to FTB.

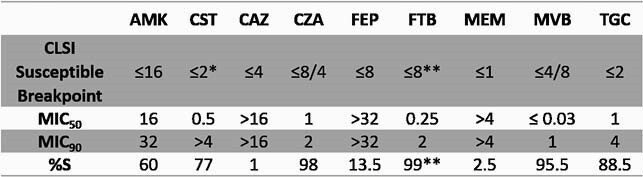

Table 1. MIC50 and MIC90 values (μg/mL) and percent susceptibility for Klebsiella pneumoniae strains (n=200). AMK, amikacin; CST, colistin; CAZ, ceftazidime; CZA, ceftazidime-avibactam; FEP, cefepime; FTB, cefepime-taniborbactam; MEM, meropenem; MVB, meropenem-vaborbactam; TGC, tigecycline. * The breakpoint for CST is intermediate, as no susceptible breakpoint is available. ** The susceptible-dose-dependent breakpoint for FEP alone was provisionally applied to FTB, where taniborbactam was fixed at 4 μg/mL. Breakpoints from CLSI M100, 31st ed, 2021.

**Conclusion:**

The addition of taniborbactam restored susceptibility to FEP in 99.0% of CRACKLE isolates studied, comparable to CZA and MVB. Taniborbactam also restored FEP activity against some MVB- and CZA-resistant strains. FTB may provide a promising therapy for CRK infections.

**Disclosures:**

**Robin Patel, MD**, **1928 Diagnostics** (Consultant)**BioFire Diagnostics** (Grant/Research Support)**ContraFect Corporation** (Grant/Research Support)**Curetis** (Consultant)**Hylomorph AG** (Grant/Research Support)**IDSA** (Other Financial or Material Support, Editor’s Stipend)**Infectious Diseases Board Review Course** (Other Financial or Material Support, Honoraria)**Mammoth Biosciences** (Consultant)**NBME** (Other Financial or Material Support, Honoraria)**Netflix** (Consultant)**Next Gen Diagnostics** (Consultant)**PathoQuest** (Consultant)**PhAST** (Consultant)**Qvella** (Consultant)**Samsung** (Other Financial or Material Support, Patent Royalties)**Selux Diagnostics** (Consultant)**Shionogi & Co., Ltd.** (Grant/Research Support)**Specific Technologies** (Consultant)**TenNor Therapeutics Limited** (Grant/Research Support)**Torus Biosystems** (Consultant)**Up-to-Date** (Other Financial or Material Support, Honoraria) **Robin Patel, MD**, BioFire (Individual(s) Involved: Self): Grant/Research Support; Contrafect (Individual(s) Involved: Self): Grant/Research Support; IDSA (Individual(s) Involved: Self): Editor’s stipend; NBME, Up-to-Date and the Infectious Diseases Board Review Course (Individual(s) Involved: Self): Honoraria; Netflix (Individual(s) Involved: Self): Consultant; TenNor Therapeutics Limited (Individual(s) Involved: Self): Grant/Research Support; to Curetis, Specific Technologies, Next Gen Diagnostics, PathoQuest, Selux Diagnostics, 1928 Diagnostics, PhAST, Torus Biosystems, Mammoth Biosciences and Qvella (Individual(s) Involved: Self): Consultant **David van Duin, MD, PhD**, **Entasis** (Advisor or Review Panel member)**genentech** (Advisor or Review Panel member)**Karius** (Advisor or Review Panel member)**Merck** (Grant/Research Support, Advisor or Review Panel member)**Pfizer** (Consultant, Advisor or Review Panel member)**Qpex** (Advisor or Review Panel member)**Shionogi** (Grant/Research Support, Scientific Research Study Investigator, Advisor or Review Panel member)**Utility** (Advisor or Review Panel member) **Vance G. Fowler, Jr., MD, MHS**, **Achaogen** (Consultant)**Advanced Liquid Logics** (Grant/Research Support)**Affinergy** (Consultant, Grant/Research Support)**Affinium** (Consultant)**Akagera** (Consultant)**Allergan** (Grant/Research Support)**Amphliphi Biosciences** (Consultant)**Aridis** (Consultant)**Armata** (Consultant)**Basilea** (Consultant, Grant/Research Support)**Bayer** (Consultant)**C3J** (Consultant)**Cerexa** (Consultant, Other Financial or Material Support, Educational fees)**Contrafect** (Consultant, Grant/Research Support)**Debiopharm** (Consultant, Other Financial or Material Support, Educational fees)**Destiny** (Consultant)**Durata** (Consultant, Other Financial or Material Support, educational fees)**Genentech** (Consultant, Grant/Research Support)**Green Cross** (Other Financial or Material Support, Educational fees)**Integrated Biotherapeutics** (Consultant)**Janssen** (Consultant, Grant/Research Support)**Karius** (Grant/Research Support)**Locus** (Grant/Research Support)**Medical Biosurfaces** (Grant/Research Support)**Medicines Co.** (Consultant)**MedImmune** (Consultant, Grant/Research Support)**Merck** (Grant/Research Support)**NIH** (Grant/Research Support)**Novadigm** (Consultant)**Novartis** (Consultant, Grant/Research Support)**Pfizer** (Grant/Research Support)**Regeneron** (Consultant, Grant/Research Support)**sepsis diagnostics** (Other Financial or Material Support, Pending patent for host gene expression signature diagnostic for sepsis.)**Tetraphase** (Consultant)**Theravance** (Consultant, Grant/Research Support, Other Financial or Material Support, Educational fees)**Trius** (Consultant)**UpToDate** (Other Financial or Material Support, Royalties)**Valanbio** (Consultant, Other Financial or Material Support, Stock options)**xBiotech** (Consultant) **Daniel D. Rhoads, MD**, **Becton, Dickinson and Company** (Grant/Research Support) **Michael Jacobs, MBBS**, **Venatorx Pharmaceuticals, Inc.** (Grant/Research Support) **Focco van den Akker, PhD**, **Venatorx Pharmaceuticals, Inc.** (Grant/Research Support) **David A. Six, PhD**, **Venatorx Pharmaceuticals, Inc.** (Employee) **Greg Moeck, PhD**, **Venatorx Pharmaceuticals, Inc.** (Employee) **Krisztina M. Papp-Wallace, Ph.D.**, **Merck & Co., Inc.** (Grant/Research Support)**Spero Therapeutics, Inc.** (Grant/Research Support)**Venatorx Pharmaceuticals, Inc.** (Grant/Research Support)**Wockhardt Ltd.** (Other Financial or Material Support, Research Collaborator) **Robert A. Bonomo, MD**, **entasis** (Research Grant or Support)**Merck** (Grant/Research Support)**NIH** (Grant/Research Support)**VA Merit Award** (Grant/Research Support)**VenatoRx** (Grant/Research Support)

